# Patients with blastic plasmacytoid dendritic cell neoplasm in pregnancy: A rare case report

**DOI:** 10.1097/MD.0000000000030622

**Published:** 2022-09-23

**Authors:** Li Zhang, Yidong Wang, Mingming Lu, Mengdan Shen, Zhao Duan

**Affiliations:** a Department of Gynecology and Obstetrics, The Second Affiliated Hospital of Xi’an Jiaotong University, Xi’an, China.

**Keywords:** Blastic plasmacytoid dendritic cell neoplasm, case report, chemotherapy, pregnancy, skin nodules

## Abstract

Previous clinical reports of patients with blastic plasmacytoid dendritic cell neoplasm (BPDCN) in pregnancy are rare.

**Patient concerns:**

The present study presents the case of 37-year-old women exhibiting third trimester with progressive painless, abdominal skin nodules.

**Interventions and outcomes:**

A 37-year-old pregnant woman with BPDCN and partial placenta previa and racket-shaped placenta. After comprehensive evaluation, the pregnancy status ends at 37 weeks and 6 days by cesarean section of lower uterus and no abnormality in the newborn.

**Lessons:**

Pregnant women diagnosed with BPDCN in the third trimester should terminate the pregnancy promptly for further treatment.

## 1. Introduction

Blastic plasmacytoid dendritic cell neoplasm (BPDCN) is an infrequent hematological malignancy derived from precursor plasmacytoid dendritic cells and the overall incidence rate is 0.04 cases per 100,000 people.^[1]^ The clinical manifestations of BPDCN of BPDCN is widely heterogeneous and was categorized as a distinct clinical entity by the World Health Organization in 2016, the disease is aggressive and with a poor prognosis.^[2,3]^ Patients with BPDCN in pregnancy are rare reported.

The most presenting sign of patients with BPDCN is cutaneous lesions.^[4]^ Other common sites of disease involvement include the bone marrow and lymph nodes^[5]^ and approximately 10% of patients will present with acute leukemia.^[6]^ The diagnosis of BPDCN is usually established via skin biopsy with immunophenotyping. Morphology combined with immunophenotype are required for the diagnosis of BPDCN, with expression of 5 following markers considered necessary: CD4, CD56, CD123, TCL1, and CD303.^[7]^

In the current case report, we present a BPDCN patient during pregnancy and termination of pregnancy immediately after diagnosis. This is a rare case as its onset and rapid progress in the third trimester, with termination of pregnancy immediately following diagnosis and then performing oncology treatment.

## 2. Case report

### 2.1. Patient concerns and diagnosis

**Figure 1.**
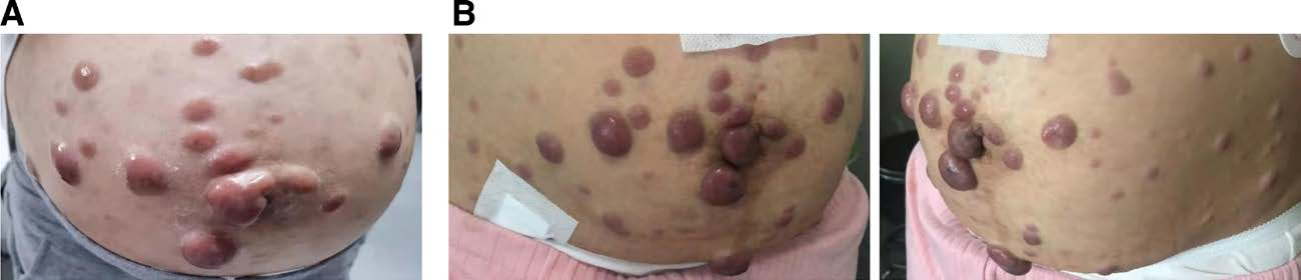
Clinical photograph of the lymphoma during pregnancy. (A) The abdominal neoplastic lesions before delivery. (B) The abdominal neoplastic lesions after delivery.

**Figure 2.**
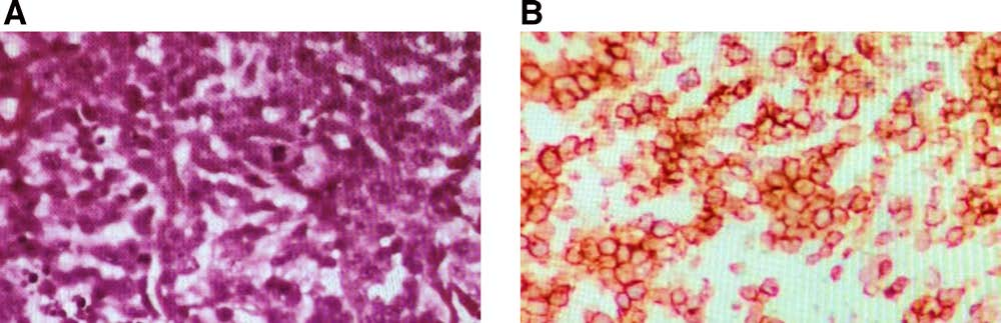
Histopathology of blastic plasmacytoid dendritic cell neoplasm. (A) Skin hematoxylin and eosin staining. (B) The neoplastic cells show immunoreactivity to CD4. Magnification × 200.

A 37-year-old woman at a gestational age of 37 weeks visited the Second Affiliated Hospital of Xi’an Jiaotong University (Shaanxi, China) in July 2021 complaining of progressive painless, multiple abdominal skin nodules (Fig. 1A) over a 3-month period, multiple skin nodules of which the maximum diameter is 2 cm. She had no relevant medical history. The initial dermatology skin nodule biopsy examination combined with immunohistochemical results (the cells typically express CD56, CD4, CD43) to support skin lymphohematopoietic system tumors, considered myeloid origin (Fig. 2).

### 2.2. Interventions and outcomes

**Figure 3.**
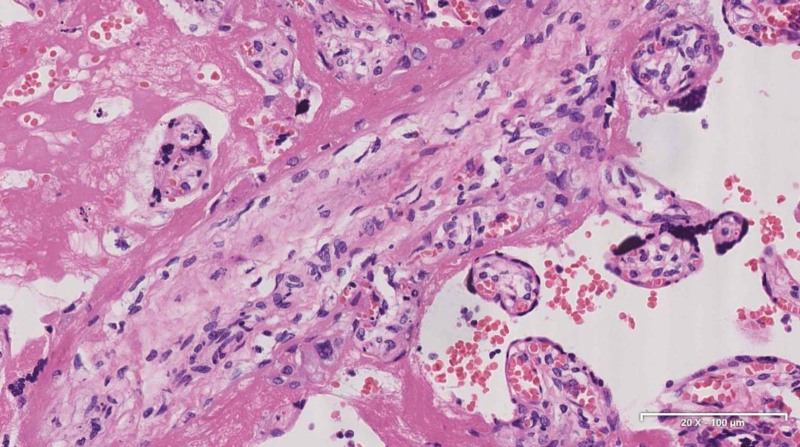
Placenta hematoxylin and eosin staining. Magnification × 200.

The pregnant woman is already in the third trimester, diagnosed at 37 weeks and 5 days of gestation and combined with partial placenta previa and racket-shaped placenta, a cesarean section was performed in Obstetrics and Gynecology. She delivered a male infant of 3520 g and measuring 50 cm, APGAR score 9/10/10, the newborn has no obvious abnormalities after being evaluated by the neonatologist. Prophylactic antibiotic therapy for cesarean section was administered. Surgery was done under epidural anesthesia without incidents, except for moderate bleeding, due to thrombocytopenia (83 × 10^9^/L) while the coagulation function of the patient was basically normal before the operation. No tumor cell infiltration of placenta pathology (Fig. 3). She did not breastfeed the newborn and no neonatal complication were reported. No severe infection was observed during the puerperal period and her symptoms relieved after childbirth, the skin nodules around the navel were smaller than before (Fig. 1B). She continued to see the hematology department 4 days later.

**Figure 4.**
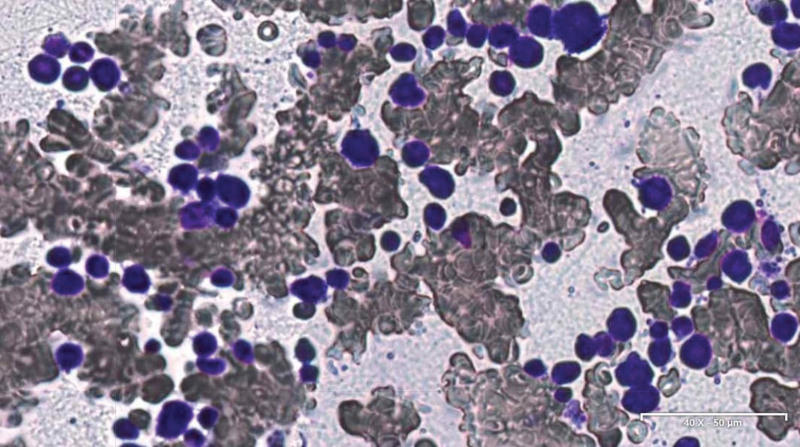
Bone marrow morphological examination (Wright-Giemsa). magnification × 200.

Immediately after cesarean section, bone marrow morphological examination was performed, the result showed bone marrow hyperplasia was significantly active, megakaryocytes and platelets were rare, and a large number of abnormal cells accounted for 88.5% (Fig. 4). Bone marrow revealed cells proliferation is active, and aberrant cells are distributed in large sheets with a CD4^ + ^CD56^ + ^CD123^ + ^CD14^ + ^ CD33^ + ^CD36^ + ^CD64^ + ^CD123^ + ^immunophenotype. Based on the clinicopathological correlation the diagnosis is blastic plasmacytoid dendritic cell neoplasm (BPDCN). Chemotherapy was initiated with CDOPE (cyclophosphamide, vincristine, cisplatin, prednisone, etoposide). After chemotherapy, the size of tumors in the patient’s abdomen was significantly reduced and there was no discomfort.

## 3. Discussion

Hematological cancers in pregnancy are rare and pose diagnostic and therapeutic challenges. Management should focus on the survival of mother, while minimizing treatment-related fetal toxic effects. Blastic plasmacytoid dendritic cell neoplasm (BPDCN) occurred rarely during pregnancy.^[8–12]^ We here perform a case of a patient who diagnosed BPDCN during pregnancy. A partial placenta previa and racket-shaped placenta was diagnosed at 37 weeks and 5 days.

The diagnosis of hematological malignancies is challenged by an overlap of the disease and gestation-related symptoms and limitations of imaging studies during pregnancy.^[13,14]^ Pregnancy itself may produce symptoms similar to those of BPDCN so that leading to a significant delay in diagnosis of patient and a higher frequency of developing to advanced disease stage. BPDCN often presents cutaneous lesions as initially symptom and subsequently progresses to bone marrow involvement and leukemic dissemination.^[15]^ In this case report, tumor biopsy was performed to confirm the disease. The pathological examination results prompt tumor of myeloid origin. Later the result of bone marrow puncture is diagnosed as BPDCN.

The management and evaluation of BPDCN during pregnancy is complex. Considerations include clearing histologic diagnosis, tumor staging and type, timing of therapy and timing of pregnancy termination. There are no large prospective studies for induction regimens in BPDCN given this is a rare disease. In the present, the primary goal is to preserve the mother’s health so as to the pregnancy termination is often advisable at early stage, allowing delivery of adequate therapy.^[13]^ Our patient was diagnosed at the 37 weeks and 5 days of pregnancy, and the disease has progressed rapidly in the past 3 months. Considering that the patient has a full-term pregnancy with partial placenta previa and racket-shaped placenta, we terminated the pregnancy as soon as possible and she delivered a healthy baby. The patient received chemotherapy immediately after termination of pregnancy.

The treatments used for induction BPDCN are based on the therapy of acute leukemia or non-Hodgkin lymphoma (NHL). Aggressive BPDCN has high relapse rate, hematopoietic stem cell transplant (HSCT) is recommended for consolidation for most eligible patients. Because of the significant toxicities associated with induction chemotherapy or transplant and the limited responses seen in the lower-intensity regimens, there is a need for development of novel agents that may provide durable responses with reduced toxicities. A number of targeted and immunomodulatory therapies that are being investigated now. Currently, the most promising data are SL-401 and CD123.^[16]^ In this case, the CDOPE (cyclophosphamide, vincristine, cisplatin, prednisone, etoposide) was used foe chemotherapy, and after treatment, the patient’s symptoms were significantly relieved.

BPDCN during pregnancy is rare. When making treatment decisions for cancer during pregnancy, it is important to consider the best treatment options for the pregnant woman balanced against the possible risks to the growing baby. In our case, the BPDCN remained diagnosed in the third trimester and the condition progresses rapidly, timely termination of pregnancy can help to slow disease progression and allow for further treatment. More information is needed for the effect of pregnancy on the clinical course and outcome of BPDCN.

## Author contributions

Conceptualization: Li Zhang.

Data curation: Li Zhang and Mengdan Shen.

Formal analysis: Li Zhang and Mingming Lu.

Supervision: Zhao Duan.

Writing—original draft: Li Zhang and Yidong Wang.

Writing—review and editing: Zhao Duan.
